# Details preserved unsupervised depth estimation by fusing traditional stereo knowledge from laparoscopic images

**DOI:** 10.1049/htl.2019.0063

**Published:** 2019-11-13

**Authors:** Huoling Luo, Qingmao Hu, Fucang Jia

**Affiliations:** 1 Research Lab for Medical Imaging and Digital Surgery, Shenzhen Institutes of Advanced Technology, Chinese Academy of Sciences, Shenzhen, People's Republic of China; 2 Shenzhen College of Advanced Technology, University of Chinese Academy of Sciences, Shenzhen, People's Republic of China

**Keywords:** stereo image processing, surgery, image reconstruction, unsupervised learning, medical image processing, phantoms, image motion analysis, convolutional neural nets, traditional stereo method, proxy disparity labels, unreliable depth measurements, confidence measure, stereo accuracy, disparity images, rectified stereo images, proxy labels, smooth depth surface, unsupervised depth estimation, traditional stereo knowledge, laparoscopic images, vision-based laparoscope surgical navigation systems, truth depth, unsupervised learning depth estimation approach, dual encoder-decoder convolutional neural network, loss function, principled mask, parallax effects, neighbourhood smoothness term, constrain neighbouring pixels, partial nephrectomy da Vinci surgery dataset, heart phantom data, Hamlyn Centre

## Abstract

Depth estimation plays an important role in vision-based laparoscope surgical navigation systems. Most learning-based depth estimation methods require ground truth depth or disparity images for training; however, these data are difficult to obtain in laparoscopy. The authors present an unsupervised learning depth estimation approach by fusing traditional stereo knowledge. The traditional stereo method is used to generate proxy disparity labels, in which unreliable depth measurements are removed via a confidence measure to improve stereo accuracy. The disparity images are generated by training a dual encoder–decoder convolutional neural network from rectified stereo images coupled with proxy labels generated by the traditional stereo method. A principled mask is computed to exclude the pixels, which are not seen in one of views due to parallax effects from the calculation of loss function. Moreover, the neighbourhood smoothness term is employed to constrain neighbouring pixels with similar appearances to generate a smooth depth surface. This approach can make the depth of the projected point cloud closer to the real surgical site and preserve realistic details. The authors demonstrate the performance of the method by training and evaluation with a partial nephrectomy da Vinci surgery dataset and heart phantom data from the Hamlyn Centre.

## 1 Introduction

Estimating the depth of a surgical site's surface is one of the key challenges in the field of computer-assisted laparoscopic surgery. This process reconstructs the intraoperative organ surface, and then, the surface can be used for registration to preoperative models derived from computed tomography or magnetic resonance image scans. To accurately predict the depth of the organ surface based on laparoscopic images or video is essential for vision-based surgical navigation systems.

In minimally invasive surgery, stereo laparoscopes have been introduced to provide surgeons with a 3D view of surgical site and to provide the fundamental input of stereo algorithms for recovering the 3D geometry without any external devices [1]. Stoyanov *et al.* [2] presented a semidense reconstruction approach for robotic assisted surgery by first identifying a set of candidate feature matches as seeds, and then, a region growing method was used to propagate disparity information around the seeds to reconstruct a semidense surface. Penza *et al.* [3] proposed two methods that followed the traditional approach of sum of absolute difference based and census transform and then refined the disparity image using super-pixel segmentation. Chang *et al.* [4] introduced a dense stereo reconstruction approach using convex optimisation cost-volume to reconstruct the model in the surgical scene. Wang *et al.* [5] developed a variational disparity estimation to minimise a global energy function over the entire image. In addition to the stereo vision-based methods, monocular laparoscope can also be taken as input to predict depth information. Mahmoud *et al.* [6] presented an extended ORB-SLAM algorithm to simultaneously reconstruct a semidense map of the surgical site and estimate the laparoscope location.

In the last few years, depth estimation using convolutional neural networks (CNNs) has shown promising results in computer vision community. Eigen *et al.* [7] treated the depth prediction as a supervised learning problem and trained a coarse-to-fine scale CNN model to estimate the depth from one monocular image. While great success of such methods built on the base of large ground truth image collections has been achieved, it is difficult to transfer this method to the laparoscopic domain. The ground truth depth or disparity data are difficult to obtain in laparoscopy, which inhibits the use of supervised methods. Garg *et al.* [8] proposed an unsupervised CNN framework to estimate a single-view depth using stereo pairs as training samples to generate an inverse warp image and minimise the projection error. Similarly, Godard *et al.* [9] proposed to train the unsupervised CNN model by enforcing left–right disparity consistency and introduced the encoder–decoder network like DispNet [10]. However, the appearance of the laparoscopic image is very different from the image in the natural scene and prevents the direct deployment of approaches from the computer vision community to laparoscope. Nevertheless, there are some research groups that have addressed the depth estimation of surgical site using deep learning methods. Ye *et al.* [11] presented a self-supervised Siamese network for depth estimation with stereo image pairs as the training examples in robotic surgery. Liu *et al.* [12] proposed a self-supervised approach with the help of coordinate transformation from a multi-view stereo method to train a two-branch Siamese network for dense depth estimation.

**Fig. 1:**
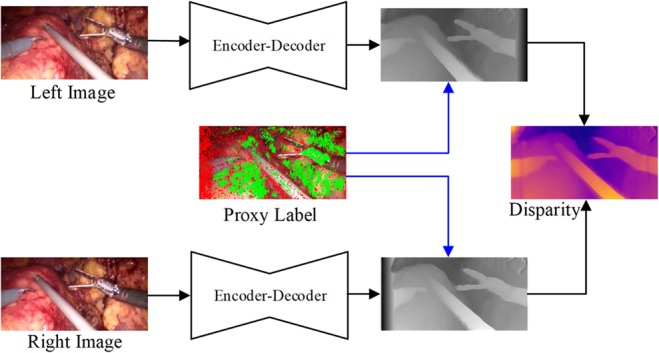
Illustration of dual encoder–decoder unsupervised depth estimation fusing traditional stereo knowledge CNN architecture

Inspired by the successful work of Tosi *et al.* [13] and to address the deficiency of ground truth data in laparoscopy, in this work, we propose a method that fuses traditional stereo vision algorithms and confidence measures to produce a highly reliable proxy label to guide the training of a dual decoder–encoder disparity prediction unsupervised CNN model. Our motivation is that there are countless stereo depth estimation methods for computer vision have been proposed during the last few decades, particularly in the field of laparoscopy, many of these methods have achieved the state-of-the-art performance. Moreover, the confidence measures which aim to detect unreliable depth measurements of the predicted depth map can be used for distilling the error estimation. The results from the traditional off-the-shelf stereo method can provide guidance for the training of the unsupervised depth estimation CNN model when laparoscopic ground truth data are not available. Fig. 1 shows the architecture of our proposed dual encoder–decoder unsupervised depth estimation CNN, which fuses traditional stereo knowledge. Proxy disparity labels are generated by standard stereo algorithms that are distilled by the confidence measure prior model training and only used during the training phase. The rectified stereo pairs are fed into the dual encoder–decoder CNN, to generate the corresponding disparity maps. To focus attention on pixels that can be seen on both views, a principled mask is computed when calculating the training loss. In addition, we introduce a neighbourhood smoothness term to constrain neighbouring pixels with similar appearances, which can generate smooth depth surface in reality. The effectiveness of our proposed unsupervised approach is demonstrated by extensive training and evaluation with a partial nephrectomy da Vinci surgery dataset and heart phantom data from Hamlyn Centre [2, 14].

## 2 Methods

Most existing laparoscopic data with ground truth are created based on phantom or ex vivo [2, 15, 16] experimental settings; however, their appearances are very different from that of the real surgical scene. Although these algorithms achieve good performance on these data, they may suffer degradation when transferred to the real surgical site. To address the above challenge, we introduce a method for depth estimation by training the model on a real surgical dataset, in an unsupervised manner, by fusing traditional stereo knowledge to guide the network training.

### 2.1 Dual encoder–decoder disparity network

**Fig. 2:**
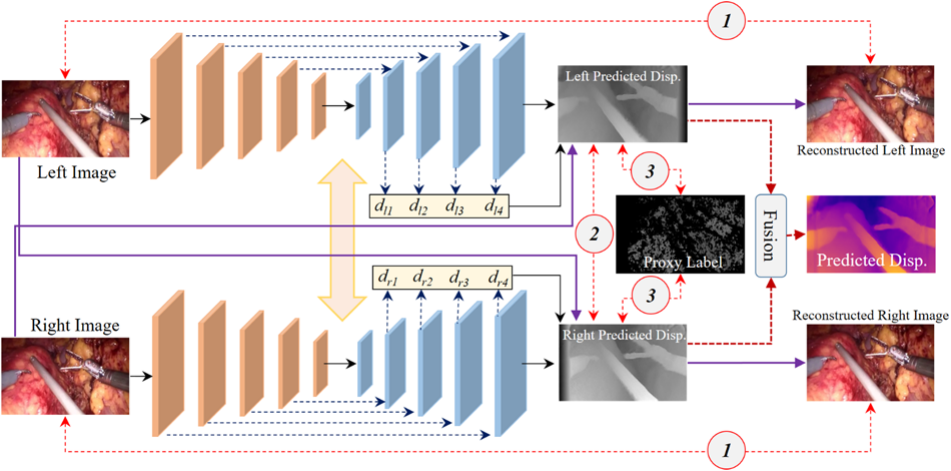
Dual encoder–decoder disparity network. The network outputs four different scales disparity maps. The final estimation of disparity is the fusion of d_l4_ and d_r4_ from the left and right part, respectively. The numbers with circle are different loss terms: ① image reconstruction loss, ② left–right disparity consistency loss, ③ proxy label loss

Our disparity estimation network architecture is shown in Fig. 2. The architecture consists of two branches for the encoder–decoder network, which has the same architecture as [9], and both branches share the weight in the training phase. The encoder part of disparity network is built upon ResNet-50 [17]. The decoder outputs four different scales disparity maps and uses skip connections from encoder's activation blocks that enable it to resolve higher resolution details.

To fully use the image reconstruction information from different views of the stereo pair, we set the left and right image as the input of the two-branch network to obtain the left-to-right and right-to-left disparity maps, respectively. Based on the predicted disparity maps and the original image, we can reconstruct corresponding left and right images. The original stereo pair and reconstructed images are constrained by photometric image reconstruction loss $L_r$ as in [9]. Furthermore, the proxy disparity label acts as a sparse term to supervise the signal to the predicted disparity maps using $L_1$ loss (see Section 2.2), which can make the estimation of disparity closer to the real scene. Finally, the predicted disparity maps of the two branches are evaluated by left–right disparity consistency loss $L_{rc}$ [9]. We believe that the stereo pair will supply more useful information for the final estimation of disparity. Therefore, the fusion of the finest scales of disparity maps is set as the output in the testing phase.

### 2.2 Proxy disparity label

**Fig. 3:**
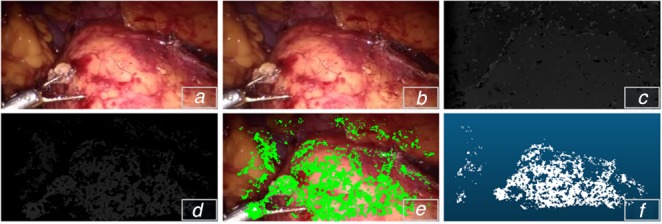
Proxy disparity label *a* Left image of stereo pair *b* Right image of stereo pair *c* Output of AD-CENSUS *d* Result of disparity distillation *e* Proxy label sampled on RGB image *f* Disparity reprojected to 3D points

To generate accurate and reliable proxy disparity labels, we adopt the conventional stereo algorithm AD-CENSUS [18], and some of the quantitative evaluation of confidence measures listed in [19] are used for distilling unreliable estimation of the disparity map. More specifically, the stereo pair is fed into the stereo algorithm to generate the disparity label while the confidence measures, including left–right consistency checking (LRC), uniqueness constraint (UC), distance to border (DB), average peak ratio (APKR), and winner margin (WM) measure, are employed to exclude the unreliable and incorrect prediction pixels of the disparity map. Fig. 3 shows the result of the distillation of the proxy disparity labels.

Given an input of stereo pair $I_l$ and $I_r$, the dual encoder–decoder network can predict the corresponding disparity maps $d_l$ and $d_r$, respectively. Proxy disparity labels $\tilde d$ can be used as a guided signal during the train of network. We define the proxy label loss term $L_p$ as

(1) $$\eqalign{L_p & =\displaystyle{1 \over {N_p}}\sum\limits_{i\comma j \in \Omega } {\left\vert {d_l\lpar i\comma \; j\rpar - \tilde d\lpar i\comma \; j\rpar } \right\vert } \cr & \quad + \left\vert {d_r\lpar i + d_l\lpar i\comma \; j\rpar \comma \; j\rpar - \tilde d\lpar i\comma \; j\rpar } \right\vert \comma \; } \eqno\lpar 1\rpar $$

where Ω denotes the non-zero pixels in proxy disparity label, and $N_p$ denotes sum of these non-zero pixels.

### 2.3 Neighbourhood smoothness loss

**Fig. 4:**
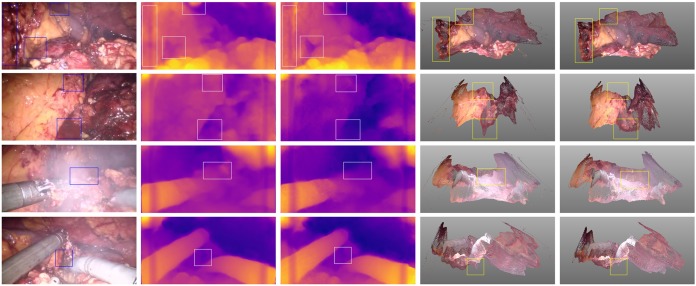
Examples of the proposed model and method of [9]. Columns from left to right: original image, disparity map of [9], disparity map of our model, point cloud of [9], point cloud of our model. The rectangle indicates the improvement of our model

From the results produced using the method [9], we observe that the depth varies even in the same tissue. The performance of these methods will suffer degradation on the surgical scene. Neighbouring pixels with similar appearance in the same tissue or organ are similar to the approximate depth or smooth surface in reality, except for the pixels on the edges of different organs or surgical tools. This phenomenon can be seen from Fig. 4 when the disparity map was projected to 3D points based on camera parameters. The rectangles in second row of Fig. 4 indicate the various depth estimation even these pixels in the same region, which results in a smooth surface after applying the neighbourhood smoothness constraint.

Consider the 4-neighborhood pixels $p_i$ and $p_j$ in the predicted disparity map. Their depth values are denoted as $D_i$ and $D_j$, respectively. We introduce the neighbourhood smoothness constraint loss [20] $L_s$ as

(2) $$L_s = \lambda \sum\limits_{\,j - i = 1} {{\left({D_i - D_j} \right)}^2{\rm e}^{ - t{\lpar f_i - f_j\rpar }^2}} \comma \; \eqno\lpar 2\rpar $$

where *λ* and *t* are positive constants to balance the weight. $f_i$ and $f_j$ refer to pixel values of $p_i$ and $p_j$ in the feature maps, respectively, which correspond to the appearance of the two pixels. More specifically, we adopt the output of convolution layers of the dual decoder–encoder network as the feature map to measure the similarity of adjacent pixels. The smoothness loss term is introduced because neighbourhood pixels that have different appearances have smaller weights, and those with similar appearances have larger weights.

### 2.4 Training loss function

The total training loss consists of photometric image reconstruction loss $L_r$, left–right disparity consistency loss $L_{rc}$, proxy label loss $L_p$, and neighbourhood smoothness constraint loss $L_s$. All of these loss terms are calculated at four different scales. Thereby, the total loss function is the combination of four loss terms of four scales *s* as

(3) $$L_{{\rm total}} = \sum\limits_{s = 1}^4 {\alpha L_r^s + \beta L_{rc}^s + \gamma L_p^s + \varphi L_s^s } \eqno\lpar 3\rpar $$

where $\alpha $, $\beta $, $\gamma $, and $\varphi $ are constants to balance the weight between different loss terms. Empirically, we set $\alpha = 1.0$, $\beta = 1.0$, $\gamma = 0.1$, and $\varphi = 0.5$ in our experiments.

#### 2.4.1 Principled masks

Due to the parallax effects of stereo pairs, some pixels near the edge of one image will not be seen on the counterpart. These pixels will degrade the performance if they are used in the loss calculation. We employ the same approach as [21] to compute the validity masks to exclude pixels that are not in both views.

#### 2.4.2 Implementation details

We use TensorFlow [22] to implement the proposed network, and train it on an NVIDIA Tesla V100 GPU with 32 Gb memory. The confidence measures listed in [19] are used to generate the proxy disparity labels prior in the training phase. The ResNet-50 backbone network is employed for the encoder part, and the decoder part consists of five deconvolutions to upsample the feature map. Skip connections are used to pass information from the encoder to the decoder part for more effective feature aggregation. The principled masks [21] are calculated during the bilinear sampling to focus the valid pixels in the final loss calculation. The batch size is set to 8. Training is performed for 50 epochs. The learning rate is initially set to 1 × 10^−4^ and varied based on training steps.

## 3 Experiments

We conduct two types of experiment to show the validity and accuracy of our proposed method and compare the results with those of the baseline method [9].

### 3.1 Dataset and evaluation

We first conduct an experiment to demonstrate the validity of detail preserving of the proposed method on in vivo data from [11], which consists of 34,240 pairs training data and 7191 pairs testing data with 384 × 192 image resolution. These images are collected in partial nephrectomy in da Vinci surgery and have been rectified. Additionally, the camera parameters are provided. As ground truth data are not available for this dataset, and the intraoperative models are not provided, we cannot use the evaluation method as computer vision community or as the method presented in [12]. A compromised evaluation method is employed in our experiment. We use a rigid confidence measurement, including LRC, UC, DB, APKR, WM and difference with median, to generate the proxy disparity maps, then we triangulate to 3D point clouds based on the camera parameters to act as the proxy ground truth data.

The second experiment is conducted based on two heart phantom data from [2, 14] to show the depth estimation accuracy of our method. The first heart phantom dataset includes 2426 frames, and the second includes 3366 frames. Both datasets have the corresponding ground truth data indexed from frame 0 to 19. The ground truth data are excluded when training the models.

**Fig. 5:**
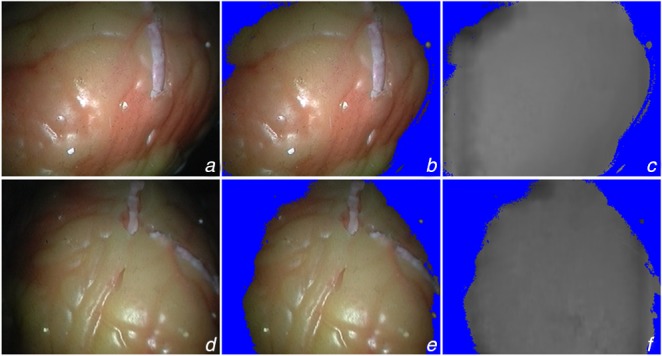
Heart phantom datasets [2, 14] and result of threshold *a–c* Extracted from Heart 1 *d–f* Extracted from Heart 2 *c* and *f* are the predicted disparity maps by our proposed method

For fair comparison with other approaches, we adopt the commonly used metrics in the laparoscope image reconstruction field, including reconstruction mean absolute errors (MAE) and root mean square error (RMSE), defined as

(4) $${\rm MAE} = \displaystyle{1 \over N}\mathop \sum \limits_{i\comma j} \left\vert {{\tilde D}_{i\comma j} - D_{i\comma j}} \right\vert \comma \; \eqno\lpar 4\rpar $$

(5) $${\rm RMSE} = \sqrt {\displaystyle{1 \over N}\mathop \sum \limits_{i\comma j} {\left({{\tilde D}_{i\comma j} - D_{i\comma j}} \right)}^2} \comma \; \eqno\lpar 5\rpar $$

where *N* is the number of non-zero point in ground truth data, and $\tilde D_{i\comma j}$ and $D_{i\comma j}$ are the predicted and ground truth depth values for pixel $\left({i\comma \; j} \right)$, respectively. The testing samples are excluded if the non-zero point of proxy ground truth is <10,000 (i.e. $N \lt 10\comma \; 000$), which can decrease the randomness in the evaluation of a particular point. For the heart phantom evaluation, we use a threshold processing to exclude the background pixels (as shown in Fig. 5).

### 3.2 Experimental results

**Fig. 6:**
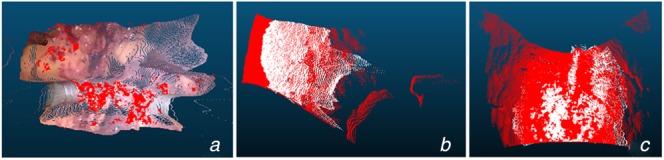
Predicted point cloud fusion with ground truth data *a* da Vinci data *b* Heart 1 *c* Heart 2 Red points indicate proxy or ground truth data, white points are the estimated points

Fig. 4 shows the qualitative result of our proposed details preserved model, especially the region indicated by rectangle. The four rows listed in Fig. 4 correspond to different scenes in laparoscopic surgery, including normal state, bleeding, smoking, and interaction with surgical tools. These images in Fig. 4 show that our method can estimate the surface more realistically. The estimated points by the proposed method compared with the da Vinci and heart phantom data are shown in Fig. 6. The red points indicate the proxy or ground truth point cloud, demonstrating the predicted point cloud can recover the truth geometry correctly.

The quantitative results are listed in Tables 1 and 2, which used the da Vinci and heart phantom dataset, respectively. For the da Vinci dataset, as we adopt the proxy ground truth for comparison, the non-zero-point count in proxy ground truth and predicted point cloud, MAE and RMSE metrics are used to compare the performance between our method and the baseline [9]. Table 2 shows the comparison of our method and others on heart phantom.

**Table 1:** Quantitative comparison on da Vinci dataset

Method	Point count	MAE, mm	RMSE, mm
monodepth [9]	19387 ± 938	10.0 ± 3.7	12.6 ± 5.8
ours	19375 ± 846	8.3 ± 3.1	10.5 ± 3.7

**Table 2:** Quantitative comparison on heart phantom data

Dataset	Method	MAE, mm	RMSE, mm
Heart 1	Godard *et al.* [9]	2.39 ± 0.62	2.99 ± 0.61
	Wang *et al.* [5]	2.16 ± 0.65	—
	Stoyanov *et al.* [2]	2.36 ± 0.92	3.88 ± 0.87
	ours	1.84 ± 0.40	2.69 ± 0.58
Heart 2	monodepth [9]	1.79 ± 0.40	2.65 ± 0.28
	Wang *et al.* [5]	2.14 ± 0.83	—
	Stoyanov *et al.* [2]	3.20 ± 1.15	4.85 ± 1.82
	ours	1.49 ± 0.41	1.90 ± 0.38

## 4 Discussion

In a real application of depth estimation, such as laparoscopic navigation, the point cloud of intraoperative surgical sites is needed. Based on this consideration, we project the predicted disparity maps to point clouds and evaluate the performance of the reconstruction MAE and RMSE between the estimated points and ground truth. Unfortunately, the ground truth depth data of real surgical site, in vivo*, * are difficult to obtain. There, we use a compromised method to evaluate the performance of our proposed model by generating the proxy ground truth disparity map employing a rigid confidence measure. Then, we triangulate to 3D point cloud to act as the proxy ground truth data for evaluation. Due to the neighbourhood smoothness loss term and the guidance of traditional information, it can be seen from Fig. 4 that our model can preserve more details, and it is closer to the actual surgical site in terms of depth prediction when projecting the disparity map to the point cloud than the baseline method [9]. The results shown in Fig. 4 (indexed by rows from top to bottom) also display four different scenes, including the normal state, haemorrhage during the operation, smoke and interaction with surgical tools. The quantitative comparison results are listed in Table 1. The results indicate that the performance of our model is obviously improved compared with the method [9], which is the basis of our network implementation.

Undoubtedly, although a more rigid confidence measure is used to generate the proxy ground truth data for evaluation, there is a certain degree of error in the method. Therefore, we conduct another experiment based on heart phantom data, which have associated ground truth, to further verify the accuracy of our proposed method. The results listed in Table 2 show that our method has a superior performance compared with that of the baseline method [9] and the other traditional methods [2, 5].

## 5 Conclusion

In this work, we proposed an unsupervised learning CNN model that fuses traditional stereo knowledge for depth estimation in laparoscope. To preserve more details and make the reconstructed point cloud closer to a real surgical site, we introduce the disparity map, which excludes the incorrect predicted pixels using confidence measures to guide the training of the dual encoder–decoder disparity network, and we employ the neighbourhood smoothness loss term to generate the same or similar appearance of pixels that are closer to the depth. We show that our model can dramatically improve the MAE and RMSE performance metric, which can render our model very useful towards real laparoscopic surgical navigation. In the future, we will test our model to predict depth in monocular videos. We also plan to evaluate our method on different datasets and compare it with state-of-the-art depth estimation methods. Moreover, the proxy disparity map will project to the 3D point cloud and guide the training of the network.

## 6 Funding and declaration of interests

This work was supported in part by Shenzhen Key Basic Science Program (JCYJ20170413162213765 and JCYJ20180507182437217), the Shenzhen Key Laboratory Program (ZDSYS201707271637577), the NSFC-Shenzhen Union Program (U1613221), and the National Key Research and Development Program (2017YFC0110903).
